# Epitope optimization of a DNA vaccine targeting SSX-2 leads to PD-1 upregulation on antigen-specific CD8 T cells and PD-L1 upregulation on tumor cells

**DOI:** 10.1186/2051-1426-1-S1-P233

**Published:** 2013-11-07

**Authors:** Brian Rekoske, Heath A Smith, Douglas G McNeel

**Affiliations:** 1 Department of Medicine, University of Wisconsin-Madison, Madison, WI, USA

## Background

Recent groups have shown that the immunological efficacy of vaccines could be increased by encoding targeted mutations that enhance the binding affinity between the encoded epitopes and the MHC-TCR complex. In this study, we sought to examine the anti-tumor efficacy of a DNA vaccine targeting SSX-2, a cancer-testis antigen (CTA) expressed by prostate tumors, containing mutations designed to enhance the affinity of the reactive epitopes for the MHC.

## Methods

A mouse sarcoma line engineered to express human HLA-A2 and SSX-2 was subcutaneously implanted into the hind flanks of C57BL/6 mice engineered to express HLA-A2 and not express mouse MHC types (HHDII-DRI mice). Mice were then treated with either a control DNA vaccine, a plasmid encoding the native SSX-2, or a plasmid encoding SSX-2 with optimized HLA-A2-binding epitopes, and tumor growth was followed.

## Results

Immunization of mice with plasmid encoding native or epitope-optimized SSX-2 elicited SSX-2 specific HLA-A2-restricted CD8+ T cells as measured by IFNγ ELISpot that were able to lyse SSX-2-expressing target cells. Despite a greater frequency of SSX-2-specific CD8+ T cells detectable following immunization with the optimized vector, immunization with the optimized vector had an inferior anti-tumor response in vivo compared with the native SSX-2 vaccine. The SSX-2-specific CD8+ T cells elicited from the optimized vaccine expressed higher levels of programmed death-1 (PD-1) compared to those elicited from the native SSX-2- vaccine, and its ligand PD-L1 was shown to be induced on the surface of tumors following immunization and could be induced in vitro by co-culturing with splenocytes from immunized animals. Ongoing studies are evaluating the efficacy of immunization with concurrent blockade of the PD-1 axis.

## Conclusions

Although much work has been directed at developing optimized cancer vaccines, our work suggests that the PD-1/PD-L1 axis serves as a dominant compensatory mechanism of resistance to this approach. Optimal next-generation DNA vaccines will likely need to be designed in such a way to either avoid or block the PD-1 regulatory pathway in order to increase their anti-tumor efficacy.

